# Exploring the Prognostic Performance of MECKI Score in Heart Failure Patients with Non-Valvular Atrial Fibrillation Treated with Edoxaban

**DOI:** 10.3390/jcm13010094

**Published:** 2023-12-23

**Authors:** Massimo Mapelli, Irene Mattavelli, Elisabetta Salvioni, Nicolò Capra, Alice Bonomi, Gaia Cattadori, Beatrice Pezzuto, Jeness Campodonico, Arianna Piotti, Alessandro Nava, Massimo Piepoli, Damiano Magrì, Stefania Paolillo, Ugo Corrà, Rosa Raimondo, Rocco Lagioia, Carlo Vignati, Roberto Badagliacca, Pasquale Perrone Filardi, Michele Senni, Michele Correale, Mariantonietta Cicoira, Marco Metra, Marco Guazzi, Giuseppe Limongelli, Gianfranco Parati, Fabiana De Martino, Francesco Bandera, Maurizio Bussotti, Federica Re, Carlo M. Lombardi, Angela B. Scardovi, Susanna Sciomer, Andrea Passantino, Michele Emdin, Caterina Santolamazza, Enrico Perna, Claudio Passino, Gianfranco Sinagra, Piergiuseppe Agostoni

**Affiliations:** 1 Centro Cardiologico Monzino, IRCCS, 20138 Milan, Italy; massimo.mapelli@ccfm.it (M.M.); irene.mattavelli@ccfm.it (I.M.); elisabetta.salvioni@ccfm.it (E.S.); nicolo.capra@ccfm.it (N.C.); alice.bonomi@ccfm.it (A.B.); beatrice.pezzuto@ccfm.it (B.P.); jeness.campodonico@cardiologicomonzino.it (J.C.); arianna.piotti@ccfm.it (A.P.); carlo.vignati@ccfm.it (C.V.); piergiuseppe.agostoni@ccfm.it (P.A.); 2 Department of Clinical Sciences and Community Health, Cardiovascular Section, University of Milan, 20122 Milan, Italy; 3 Unità Operativa Cardiologia Riabilitativa, IRCCS Multimedica, 20138 Milan, Italy; 4 University of Trieste, 34127 Trieste, Italy; nava.alessandro.14@gmail.com; 5 Clinical Cardiology, IRCCS, Policlinico San Donato, 20097 San Donato Milanese, Italy; massimo.piepoli@unimi.it; 6 Department Biomedical Sciences for Health, University of Milan, 20122 Milan, Italy; francesco.bandera@unimi.it; 7 Department of Clinical and Molecular Medicine, Azienda Ospedaliera Sant’Andrea, “Sapienza” Università degli Studi di Roma, 00185 Rome, Italy; damiano.magri@uniroma1.it; 8 IRCCS SDN, Istituto di Ricerca, 80143 Napoli, Italy; paolilloste@gmail.com; 9 Department of Advanced Biomedical Sciences, “Federico II” University, 80138 Napoli, Italy; pasquale.perronefilardi@unina.it; 10 Cardiology Department, Istituti Clinici Scientifici Maugeri, IRCCS, Veruno Institute, 28010 Veruno, Italy; ugo.corra@icsmaugeri.it; 11 Divisione di Cardiologia Riabilitativa, Istituti Clinici Scientifici Maugeri, IRCCS, 21049 Tradate, Italy; rosa.raimondo@icsmaugeri.it; 12 UOC Cardiologia di Riabilitativa, Mater Dei Hospital, 70125 Bari, Italy; roccolagioia@cbhspa.it; 13 Dipartimento di Scienze Cliniche, Internistiche, Anestesiologiche e Cardiovascolari, “Sapienza”, Rome University, 00185 Rome, Italy; roberto.badagliacca@uniroma1.it (R.B.); susanna.sciomer@uniroma1.it (S.S.); 14 Cardiology Unit, Cardiovascular Department, ASST Papa Giovanni XXIII, 24127 Bergamo, Italy; msenni@asst-pg23.it; 15 Department of Cardiology, University of Foggia, 71122 Foggia, Italy; michele.correale@libero.it; 16 Poliambulatorio San Gaetano, 36016 Thiene, Italy; mariantonietta.cicoira@univr.it; 17 Institute of Cardiology, Department of Medical and Surgical Specialities, Radiological Sciences, and Public Health, University of Brescia, 25121 Brescia, Italy; metramarco@libero.it (M.M.); lombardi.carlo@aliceposta.it (C.M.L.); 18 Dipartimento di Scienze Biomediche per la Salute, Ospedale San Paolo, Università Degli Studi di Milano, 20122 Milan, Italy; marco.guazzi@unimi.it; 19 Cardiologia SUN, Ospedale Monaldi (Azienda dei Colli), Seconda Università di Napoli, 80138 Napoli, Italy; limongelligiuseppe@libero.it; 20 Istituto Auxologico Italiano, IRCCS, 20138 Milan, Italy; gianfranco.parati@unimib.it; 21 Department of Medicine and Surgery, University of Milano-Bicocca, 20126 Milan, Italy; 22 Unità Funzionale di Cardiologia, Casa di Cura Tortorella, 84124 Salerno, Italy; fabiana.demartino@hotmail.it; 23 Cardiology University Department, IRCCS Policlinico San Donato, 20097 Milan, Italy; 24 Cardiac Rehabilitation Unit, Istituti Clinici Scientifici Maugeri, IRCCS, Scientific Institute of Milan, 20138 Milan, Italy; maurizio.bussotti@icsmaugeri.it; 25 Cardiology Division, Cardiac Arrhythmia Center and Cardiomyopathies Unit, San Camillo-Forlanini Hospital, 00152 Rome, Italy; federicare@scamilloforlanini.rm.it; 26 Cardiology Division, Santo Spirito Hospital, 00193 Rome, Italy; ab.scardovi@libero.it; 27 Division of Cardiology, Istituti Clinici Scientifici Maugeri, IRCCS, Institute of Bari, 70124 Bari, Italy; andrea.passantino@icsmaugeri.it; 28 Health Science Interdisciplinary Center, Scuola Superiore Sant’Anna, 56127 Pisa, Italy; emdin@ftgm.it (M.E.); passino@ftgm.it (C.P.); 29 Fondazione Toscana Gabriele Monasterio, 56124 Pisa, Italy; 30 Dipartimento Cardio-Toraco-Vascolare, Ospedale Cà Granda-A.O. Niguarda, 20162 Milan, Italy; caterina.santolamazza@ospedaleniguarda.it (C.S.); enrico.perna@ospedaleniguarda.it (E.P.); 31 Cardiovascular Department, “Azienda Sanitaria Universitaria Giuliano-Isontina”, 34100 Trieste, Italy; gianfranco.sinagra@asugi.sanita.fvg.it

**Keywords:** NVAF, heart failure (HF), prognosis, cardiopulmonary exercise testing (CPET), atrial fibrillation, edoxaban, DOACs, anticoagulants

## Abstract

Introduction: Risk stratification in heart failure (HF) is essential for clinical and therapeutic management. The Metabolic Exercise test data combined with Cardiac and Kidney Indexes (MECKI) score is a validated prognostic model for assessing cardiovascular risk in HF patients with reduced ejection fraction (HFrEF). From the validation of the score, the prevalence of HF patients treated with direct oral anticoagulants (DOACs), such as edoxaban, for non-valvular atrial fibrillation (NVAF) has been increasing in recent years. This study aims to evaluate the reliability of the MECKI score in HFrEF patients treated with edoxaban for NVAF. Materials and Methods: This study included consecutive outpatients with HF and NVAF treated with edoxaban (*n* = 83) who underwent a cardiopulmonary exercise test (CPET). They were matched by propensity score with a retrospective group of HFrEF patients with NVAF treated with vitamin K antagonists (VKAs) from the MECKI score registry (*n* = 844). The study endpoint was the risk of cardiovascular mortality, urgent heart transplantation, or Left Ventricle Assist Device (LVAD) implantation. Results: Edoxaban patients were treated with a more optimized HF therapy and had different clinical characteristics, with a similar MECKI score. After propensity score, 77 patients treated with edoxaban were successfully matched with the MECKI-VKA control cohort. In both groups, MECKI accurately predicted the composite endpoint with similar area under the curves (AUC = 0.757 vs. 0.829 in the MECKI-VKA vs. edoxaban-treated group, respectively, *p* = 0.452). The two populations’ survival appeared non-significantly different at the 2-year follow-up. Conclusions: this study confirms the prognostic accuracy of the MECKI score in HFrEF patients with NVAF treated with edoxaban, showing improved predictive power compared to VKA-treated patients.

## 1. Introduction

Risk stratification in heart failure (HF) is crucial for clinical and therapeutic management. Due to the complexity of the disease, a multiparametric approach involving several variables is the best method to stratify prognosis in HF patients [1,2]. The Metabolic Exercise test data combined with Cardiac and Kidney Indexes (MECKI) score is a validated prognostic model to assess the risk of cardiovascular mortality, urgent heart transplantation, and Left Ventricle Assist Device (LVAD) implantation at two years in patients with HF and reduced ejection fraction (HFrEF) [3]. According to ESC heart failure guidelines, it is one of the three suggested scores for a proper HF prognostic assessment [2]. The MECKI score integrates cardiopulmonary exercise test (CPET) parameters with easy-to-obtain variables: hemoglobin (Hb), serum sodium (Na^+^), kidney function by means of Modification of Diet in Renal Disease (MDRD) equation, left ventricle ejection fraction (LVEF), peak oxygen consumption peak VO_2_, (% pred), and ventilation/carbon dioxide production (VE/VCO_2_) slope. Non-valvular atrial fibrillation (NVAF) is a common feature in HF patients [4,5]. In the original MECKI score population [3], 17% of patients were AF subjects treated with vitamin K antagonists (VKAs). Since then, direct oral anticoagulants (DOACs) were introduced as alternative VKAs and have now emerged as the preferred choice. Therefore, the prevalence of HF patients treated with DOACs is rapidly increasing.

The aim of this study was to assess the reliability of the prognostic evaluation using the MECKI score also in HFrEF patients treated with edoxaban for NVAF. To avoid bias related to different DOACs, we limited this first study to one molecule.

## 2. Materials and Methods

We prospectively enrolled consecutive outpatients with HF and NVAF treated with edoxaban undertaking a maximal ramp-protocol CPET at the HF Unit of Centro Cardiologico Monzino, IRCCS, Milan (Italy) from November 2018. 

This population was matched by propensity score with a retrospective group of HFrEF patients with NVAF treated with VKAs belonging to the MECKI score registry. Each patient also underwent an echocardiography for LVEF (Simpson’s method) and a blood sampling for Hb, MDRD, and Na^+^. The MECKI score registry includes patients with a history of HFrEF, enrolled and prospectively followed in 27 Italian HFrEF centers participating in the Metabolic Exercise Cardiac Kidney Index (MECKI) score research group [3]. Inclusion criteria for this registry were history of HF (NYHA functional class I-IV, stage B and C of ACC/AHA classification) and previous documentation of reduced EF (<40%), unchanged HF medications for at least three months, execution of a maximal CPET, no major CV treatment or intervention scheduled. Exclusion criteria were history of pulmonary embolism, moderate-to-severe aortic and mitral stenosis, pericardial disease, severe obstructive lung disease, exercise-induced angina, and significant ECG alterations or presence of any clinical comorbidity interfering with exercise performance [6]. At enrollment, clinical history and therapy information were recorded, and then physical examination, laboratory analyses, ECG, transthoracic echocardiography, and CPET were performed, as previously described [3].

### 2.1. Cardiopulmonary Exercise Test and Echocardiography

All cardiopulmonary exercise tests (CPETs) were conducted using a stationary ergospirometer (Quark PFT Cosmed, Rome, Italy) in conjunction with an electronically braked cycle ergometer. Prior to this study, all patients had already undergone CPETs in our laboratory, considered as a familiarization test. We implemented a progressively increasing workload exercise protocol (ramp) designed to achieve peak exercise within approximately 10 min [7]. Unless interrupted by clinical events, CPETs were terminated when subjects reported reaching their maximal effort. We conducted a breath-by-breath analysis of expiratory gases and ventilation. PeakVO_2_ expressed as a percentage of predicted value (VO_2_%) was calculated according to Hansen et al. [8]. The VE/VCO_2_ slope was determined as the slope of the linear relationship between VE and VCO_2_ starting from 1 min after the initiation of loaded exercise and continuing until the end of the isocapnic buffering period [9]. VE/VCO_2_% of predicted was calculated according to Salvioni et al. [10]. It is important to note that all CPETs were carried out on separate days from other exercise efforts. The LVEF was assessed during a standard transthoracic echocardiography by means of the Simpson’s rule, as previously described [11].

### 2.2. Anticoagulation Therapy

The anticoagulant therapy (edoxaban or VKAs) was prescribed as per clinical indications [12]. In the retrospective cohort, the VKA dose was adjusted according to the international normalized ratio (target: 2–3). In the prospective cohort of patients treated with edoxaban, the daily dose was reduced (from 60 to 30 mg) in the presence of any of the following criteria: moderate renal impairment (creatinine clearance 30–50 mL/min), body weight of 60 kg or less, or concomitant use of potent P-glycoprotein inhibitors (such as erythromycin, cyclosporine, dronedarone, quinidine, or ketoconazole).

### 2.3. Statistical Analysis

Continuous variables are presented as means ± standard deviation or median and (interquartile range) as appropriate, while categorical variables are expressed as absolute numbers and percentages. In order to identify two homogeneous groups, a 1-to-2 propensity score matching was conducted, using the following variables: ischemic etiology, age, gender, BMI, creatinine, LVEF, Hb, Na+, MDRD, VE/VCO_2_ slope, and peak VO_2_ (%). Unpaired *t*-test or Kruskal–Wallis test was employed to assess difference between the two matched groups for continuous variables, while chi-square test or Fisher’s exact test was performed for analyses involving categorical variables. The study endpoint was the composite of CV death, urgent heart transplantation, or LVAD implantation analyzed at 2 years.

Receiver operating characteristic (ROC) curves were generated, and the calculation of the area under the ROC curve (AUC) allowed us to evaluate the MECKI score’s ability to predict the primary endpoint at 2 years in each of the matched groups. Cumulative survival function was estimated using the Kaplan–Meier method, and the log-rank test was used to compare the survival curves. *p*-values less than 0.05 were considered statistically significant. All analyses were carried out using SAS v9.4 (SAS Institute, Cary, NC, USA).

This study was approved by IEO-Centro Cardiologico Monzino Ethical committee (R986/19-CCM 1036). 

## 3. Results

Eighty-three patients with HF and NVAF treated with edoxaban were enrolled (prospective cohort). We used as a control group a multicenter retrospective cohort of VKA-treated HFrEF patients with AF (MECKI-VKA, *n* = 844) already included in the whole MECKI score population (*n* = 7800 at September 2023). Of note, in both groups, therapy was set by the referring physician and considered optimized according to guidelines available when patients were recruited.

After propensity score 1:2 adjustment, 77 patients were successfully matched with the MECKI-VKA control group (*n* = 154). Table 1 shows the main characteristics of the four groups considered. Importantly, edoxaban patients were treated with a more optimized HF therapy (i.e., sacubitril/valsartan was present in 64.9% vs. 2.6% in the MECKI-VKA-matched group). Nevertheless, the risk assessed using the MECKI score was similar in the two matched groups (6.8 vs. 6.6% in the MECKI-VKA vs. edoxaban, respectively, *p* = 0.60).

**Figure 1:**
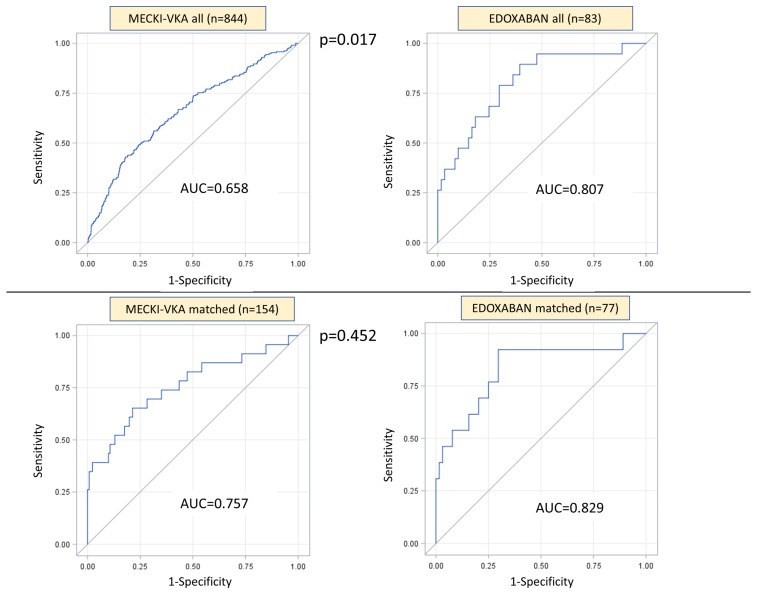
MECKI score performance evaluation according to area under the curve (AUC) at two-year follow-up. Upper panel: comparison between the retrospective cohort (MECKI-VKA, left) and the prospectively enrolled edoxaban-treated population (right). Lower panel: comparison between the two propensity-score-matched populations (left: MECKI-VKA; right: edoxaban). Study endpoint: cardiovascular death, urgent heart transplantation, or Left Ventricle Assist Device implantation.

The prognostic power of the MECKI score evaluated using the AUC at the 2-year follow up in the edoxaban-treated group was higher than that of the entire retrospective MECKI-VKA population (0.8067 vs. 0.6576, respectively, *p* = 0.017) (Figure 1, top panel).

After propensity score, we recorded 23 (15%) cardiovascular events in the MECKI-VKA-matched population and 13 (17%) in the edoxaban population (*p* = 0.700), within 2 years from enrolment data. In this case, the MECKI score performed similarly in predicting the outcome in the two groups (AUC of MECKI-VKA-matched population 0.757, AUC edoxaban group 0.829, *p* = 0.452) (Figure 1, bottom panel). 

**Figure 2:**
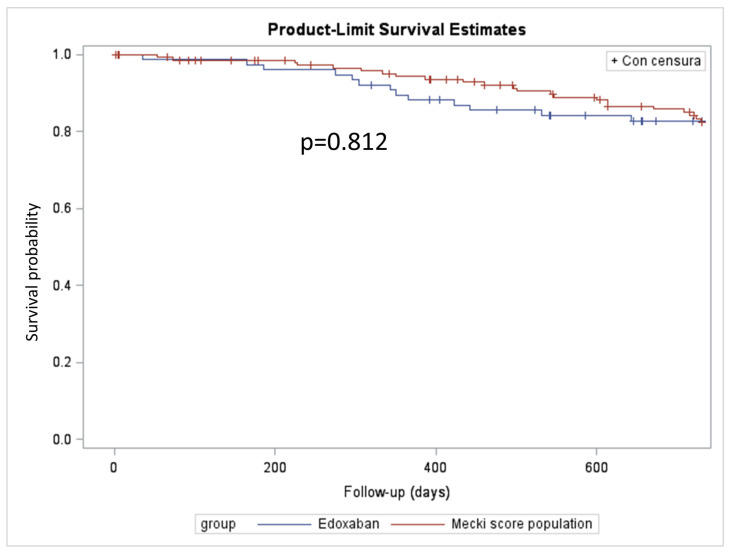
Survival free from urgent heart transplantation or Left Ventricle Assist Device implantation analysis. Kaplan–Meier curves of the two propensity-score-matched populations. Blue line: edoxaban-treated, red line: VKA-treated.

In terms of prognosis, the two populations appeared non-significantly different at the 2-year follow-up, as shown by Kaplan–Meier analysis (Figure 2).

## 4. Discussion

The main outcome of this study is the confirmation of the MECKI score prognostic accuracy in a prospective population of HFrEF patients with NVAF treated with a DOAC (edoxaban). As previously described, NVAF is a frequent comorbidity in HfrEF, being present in about one quarter of the patients [13] and representing a marker of HF severity, i.e., in patients who more need a precise prognosis assessment. NVAF, however, is not independently associated with a worst outcome, at least in HfrEF. Indeed, in a previous trial by the MECKI score research group [14] including 3447 patients (595 with NVAF), Paolillo et al. demonstrated how NVAF was associated with an increased risk of death and heart transplant compared to sinus rhythm subjects, but this was no longer true after a multivariate adjustment analysis. Similarly, in a population-based trial on a sizable population of HF patients, NVAF was not independently related to a worst prognosis in the HfrEF subgroup [13]. 

In the current study, we confirm the capability of the MECKI score to assess CV risk also in NVAF patients treated with VKA, even if with a lower performance with respect to the general population included in the original study [3] (AUC 0.658 vs. 0.789, respectively). The reasons behind this finding are unknown, although a VKA-related iron deficiency might play a role. Indeed, since Hb is one of the six parameters generating the MECKI score, anemia per se should not be the cause of the relatively poor performance of the score in VKA-treated NVAF patients. Another possible explanation could be that, even in presence of a history of NVAF, the heart rhythm at the moment of the enrollment (sinus rhythm vs. atrial fibrillation) may affect the MECKI values (i.e., both at LVEF and the variables obtained during CPET). To confirm this thesis, the AUC values obtained in the edoxaban population are significantly higher (Figure 1). In this group of patients, the presence of likely many more individuals with paroxysmal atrial fibrillation is plausible, given the nearly doubled prevalence of amiodarone therapy (60% of patients vs. 31% of the MECKI-VKA group) and the lower prevalence of digitalis therapy (10% of patients vs. 38%) (Table 1).

There is no doubt of the role of anticoagulation in reducing the thromboembolic risk in NVFA, and this is especially true in the presence of a concomitant HF, given the increased risk of thrombosis in this population [15]. DOACs have emerged as a reliable alternative to VKAs in NVAF in multiple randomized controlled trials [16,17,18], showing a better risk/efficacy profile compared to VKAs. Since the high prevalence of NVAF in HFrEF patients and given the need to assess the prognosis of these patients with risk scores, the confirmation of the prognostic accuracy of the MECKI score in this prospective cohort of patients treated with edoxaban (AUC 0.807) is reassuring. The causes of the improved prognostic power of the MECKI score in NVAF patients treated with edoxaban are unknown, although a better iron profile may be hypostatized.

Accordingly, due to the high heterogeneity of the two populations, mostly related to the different period of enrolment (MECKI-VKA was enrolled before 2019), an adjustment for the main potential confounding factors was warranted. After propensity score matching, we demonstrated a similar good performance of the MECKI score in the two matched populations (Figure 1, lower panel). 

The high incidence of cardiovascular events in the group of HFrEF treated with edoxaban (17% at 2 years) confirms the severity of the disease even in presence of an optimal HF medical therapy (Table 1). This is also shown by the metabolic values obtained at CPET. Specifically, we noted a significant exercise limitation with a relevant peak VO_2_ reduction (56.07 ± 14.32%) and an increased value of VE/VCO_2_ slope (38.0[33.5;43.4]), with such a combination of the two parameters being related to a particularly bad prognosis. The background HF therapy of the two matched groups (see Table 1) reflects the period of enrollment of the patients, with a higher prescription of sacubitril/valsartan and a lower prescription of digitalis in the prospective cohort compared to MECKI-VKA-matched patients. The comparable risk profiles between the two matched study populations, as indicated by similar MECKI scores despite a more optimized therapy, may be attributed to the fact that at the time of enrollment, the more recent population (edoxaban-treated) had already reached clinical improvements, owing to the benefits of novel HF therapies. Consequently, through propensity score matching, these patients were effectively paired with those who had a lower baseline risk in the older MECKI-VKA cohort. The dynamic nature of HFrEF as a chronic disease with many different possible trajectories (as, for example, shown in the category of “improved EF” patients [19]) is a particularly interesting topic in the field of prognostic assessment, and underlines the need of multiple re-evaluations [20].

### Limitations

The following limitations need to be acknowledged.

One of the primary limitations of this study is the temporal difference between the two groups, with the edoxaban-treated patient group belonging to a more recent prospective cohort, while the VKA group belongs to an older retrospective one. This discrepancy could have influenced the results, considering the evolution of HF therapies over time. Second, the relatively small sample size of the edoxaban-treated patient group may impact the generalizability of the results and does not allow the detection of possible differences between males and females. Further studies with larger samples are needed to confirm the findings. Third, the prospective cohort of this study was conducted at a single center, which may limit its representativeness for other populations and healthcare facilities. Additional multicenter studies are needed to confirm the results. Fourth, we do not have access to the iron status of the two populations, which would confirm the different roles of the two drugs in iron deficit. Fifth, comprehensive records capturing the exact AF status at baseline were not available. While our data suggest potential trends, especially with respect to medication usage (e.g., higher amiodarone use in the edoxaban-treated group, indicative of a possible higher prevalence of paroxysmal AF), the lack of specific baseline AF classifications remains a constraint. Finally, this study was conducted before the large-scale use of Sodium-Glucose Co-Transporter 2 Inhibitor, so the results might be different also considering the impact of these drugs in HF prognosis and on anemia.

## 5. Conclusions

NVAF-HF-edoxaban-treated patients are among the HF patients with the greatest severity. In these patients, the MECKI score maintains its strong prognostic capacity at 2 years. 

## Figures and Tables

**Table 1:** Main characteristics of the two populations considered before and after propensity score matching. Green columns: pre match comparison; Orange columns: comparison after propensity score matchng.

	Pre-Match			2:1 Propensity Score Matching		
Variable	MECKI-VKA Total (*n* = 844)	Edoxaban (*n* = 83)	*p*-Value	MECKI-VKA Matched (*n* = 154)	Edoxaban Matched (*n* = 77)	*p*-Value
**Ischemic etiology (*n* %)**	316 (38.3%)	36 (45.6%)	0.20574	66 (43%)	35 (46%)	0.707
Females (*n* %)	144 (17.1%)	10 (11.9%)	0.226	17 (11%)	8 (10%)	0.881
**Males (*n* %)**	700 (82.9%)	74 (88.1%)		137 (89%)	69 (90%)	
**Age (years)**	69 (61;76)	73.5 (66.5;80)	*0.0001*	71 (64;78)	72 (65;80)	0.491
**BMI (kg/m^2^)**	26.58 (24.09;29.63)	25.97 (24.18;29.18)	0.562	27.26 ± 4.06	26.84 ± 3.93	0.460
**LVEF (%)**	34.14 ± 10.74	34.23 ± 10.26	0.941	33.3 ± 10.1	33.9 ± 9.9	0.703
**Hemoglobin (m/dL)**	13.47 ± 1.76	13.75 ± 2.02	0.168	13.8 ± 1.8	13.8 ± 2.0	0.878
**Creatinine (mg/dL)**	1.19 (0.98;1.50)	1.29 (1.04;1.61)	*0.012*	1.25 (1.03;1.6)	1.28 (1.04;1.58)	0.795
**MDRD (mL/min/1.73 m^2^)**	63.5 (47.7;79.2)	57.5 (43.86;70.03)	*0.0140*	58.8 (45.0;74.1)	58.1 (44.8;71.6)	0.804
**Na+ (mmol/L)**	139.22 ± 3.53	140.18 ± 3.14	*0.0171*	140.2 ± 3.3	140.1 ± 3.1	0.779
Peak VO_2_ (mL/min)	989 (743.2;1223.8)	1081 (862;1318)	*0.031*	1018 (791;1260)	1081 (862;1385)	0.458
Peak VO_2_ (mL/min/kg)	12.30 (10;15.13)	14.1 (10.9;15.8)	*0.035*	13.2 (10.2;16.0)	14.1 (10.9;15.9)	0.369
**Peak VO_2_ (% pred)**	50.42 (40.81;62.13)	57.35 (45.7;64.03)	*0.009*	55.65 ± 18.18	56.07 ± 14.32	0.858
**VE/VCO_2_ slope**	34 (29;40)	38.3 (33.9;43.8)	*<0.0001*	37.32 (32;45)	38.0 (33.5;43.4)	0.517
**VE/VCO_2_ slope (% pred)**	127 (110;149)	143 (126;159)	*<0.0001*	137 (119;168)	142 (125;157)	0.5303
ACE Inhibitors (*n* %)	531 (62.9%)	18(22.5%)	*<0.0001*	94 (61%)	18 (23.4%)	*<0.0001*
AT1 inhibitors (*n* %)	182 (21.9%)	5 (6.3%)	*<0.001*	33 (21.7%)	5 (6.5%)	*0.003*
Allopurinol (*n* %)	276 (32.7%)	22 (27.5%)	0.342	58 (37.7%)	21 (27.3%)	0.117
Amiodarone (*n* %)	253 (30.8%)	49 (61.3%)	*<0.0001*	39 (25.8%)	47 (61%)	*<0.0001*
Antiplatelets (*n* %)	159 (18.8%)	35 (43.8%)	*<0.0001*	29 (18.8%)	34 (44.2%)	*<0.0001*
Betablockers (*n* %)	708 (83.9%)	70 (87.5%)	0.397	126 (81.8%)	68 (88.3%)	0.205
Digitalis (*n* %)	317 (38.2%)	8 (10%)	*<0.0001*	63 (41.4%)	8 (10.4%)	*<0.0001*
Diuretics (*n* %)	757 (89.7%)	71 (88.8%)	0.782	141 (91.6%)	68 (88.3%)	0.428
Sacubitril/Valsartan (*n* %)	26 (3.1%)	52 (61.9%)	*<0.0001*	4 (2.6%)	50 (64.9%)	*<0.0001*
MRA (*n* %)	537 (63.6%)	57 (71.3%)	0.174	103 (66.9%)	55 (71.4%)	0.484
Statin	332 (39.3%)	48 (60%)	*<0.0001*	71 (46.1%)	46 (59.7%)	0.484
Follow-up (days)	1428.5 (566;2614)	818 (588.5;1070)	*<0.0001*	1181.5 (545;2312)	825 (585;1083)	*0.001*
MECKI score	0.068 (0.026;0.15)	0.066 (0.029;0.175)	0.606	0.08 (0.03;0.17)	0.06 (0.03;0.17)	0.7981

BMI: body mass index; LVEF: left ventricular ejection fraction; MDRD: modification of diet in renal disease; Na+: sodium; Peak VO_2_: oxygen uptake at peak exercise; VE/VCO_2_ slope: ventilation vs. CO_2_ production relationship slope; ACE: angiotensin converting enzyme; AT1: angiotensin II type 1; MRA: mineralocorticoid receptor antagonist. **Bold** identifies variables used for propensity score matching.

## Data Availability

Database available on request on www.zenodo.org.

## Appendix A

Other participants to the MECKI score group to be acknowledged:

- Centro Cardiologico Monzino, IRCCS, Milan, Italy: Stefania Farina, Beatrice Pezzuto, Anna Apostolo, Pietro Palermo, Mauro Contini, Paola Gugliandolo, Umberto Attanasio
- Department of Clinical and Molecular Medicine, Azienda Ospedaliera Sant’Andrea, “Sapienza” Università degli Studi di Roma, Roma, Italy: Giovanna Gallo
- Dipartimento di Scienze Cardiovascolari, Respiratorie, Nefrologiche, Anestesiologiche e Geriatriche, “Sapienza”, Rome University, Rome, Italy: Federica Moscucci
- Department of Cardiology, Heart Failure and Heart Transplant Unit, Azienda Ospedaliera Papa Giovanni XXIII, Bergamo, Italy: Anita Iorio
- UOC Cardiologia, G da Saliceto Hospital, Piacenza, Italy: Geza Halasz, Bruno Capelli, Simone Binno
- Cardiologia SUN, Ospedale Monaldi (Azienda dei Colli), Seconda Università di Napoli, Napoli: Giuseppe Pacileo, Fabio Valente, Rossella Vastarella
- Cardiovascular Department, Ospedali Riuniti and University of Trieste, Trieste, Italy: Denise Zaffalon, Cosimo Carriere, Marco Masè, Marco Cittar, Andrea Di Lenarda; Maddalena Rossi; Emanuele Pestrin
- Istituto Auxologico Italiano, Milan, Italy: Sergio Caravita, Elena Viganò
- Cardiac Rehabilitation Unit, Istituti Clinici Scientifici Maugeri, Scientific Institute of Milan, Milan, Italy: Giovanni Marchese
- Cardiology Division, Santo Spirito Hospital, Roma, Italy: Roberto Ricci, Luca Arcari
- Division of Cardiology, Istituti Clinici Scientifici Maugeri, Institute of Cassano Murge, Bari, Italy: Domenico Scrutinio
- U.O. Cardiologia, S. Chiara Hospital, Trento, Italy: Elisa Battaia, Michele Moretti
- Ospedali Riuniti, Ancona, Italy: Maria Vittoria Matassini; Matilda Shkoza
- UOSD di Cardiologia/UTIC dell’IRCCS Neurolesi di Messina: Roland Herberg
- Federico II hospital, Napoli, Italy: Antonio Cittadini, Andrea Salzano, Alberto Marra
- ISMETT, Palermo, Italy: Eluisa Lafranca
- Cardiovascular Rehabilitation Unit, Buccheri La Ferla Fatebenefratelli Hospital, Palermo, Italy: Giuseppe Vitale
- Clinica Hildebrand Centro di riabilitazione Brissago, Switzerland: Davide Girola
